# Compound eye and ocellar structure for walking and flying modes of locomotion in the Australian ant, *Camponotus consobrinus*

**DOI:** 10.1038/srep22331

**Published:** 2016-03-15

**Authors:** Ajay Narendra, Fiorella Ramirez-Esquivel, Willi A. Ribi

**Affiliations:** 1 Department of Biological Sciences, Macquarie University, Sydney, NSW 2109, Australia; 2 Research School of Biology, The Australian National University, Canberra, ACT 2601, Australia; 3 Department of Biology, University of Lund, Lund S-22362, Sweden

## Abstract

Ants are unusual among insects in that individuals of the same species within a single colony have different modes of locomotion and tasks. We know from walking ants that vision plays a significant role in guiding this behaviour, but we know surprisingly little about the potential contribution of visual sensory structures for a flying mode of locomotion. Here we investigate the structure of the compound eye and ocelli in pedestrian workers, alate females and alate males of an Australian ant, *Camponotus consobrinus*, and discuss the trade-offs involved in optical sensitivity and spatial resolution. Male ants have more but smaller ommatidia and the smallest interommatidial angles, which is most likely an adaptation to visually track individual flying females. Both walking and flying forms of ants have a similar proportion of specialized receptors sensitive to polarized skylight, but the absolute number of these receptors varies, being greatest in males. Ocelli are present only in the flying forms. Each ocellus consists of a bipartite retina with a horizon-facing dorsal retina, which contains retinula cells with long rhabdoms, and a sky-facing ventral retina with shorter rhabdoms. We discuss the implications of these and their potential for sensing the pattern of polarized skylight.

Division of labour is one of the defining features of eusociality with fascinating and far-reaching consequences such as the production of specialized castes physiologically and behaviourally adapted to fulfilling specific roles. In ant societies, this division of labour extends beyond the type of task carried out and reproductive status, it also determines the type of locomotion, pedestrian or aerial, and therefore the kinds of visual information required by different castes. Workers are sterile females that are exclusively pedestrian and carry out all the day-to-day foraging and nest maintenance activities. In contrast, alate females are fertile and are winged; they fly out of the nest for mating, following which they shed their wings, and become pedestrian. They then start a new nest and remain for the rest of their lives within the dark confines of the nest chambers where vision plays no role. Thus, alate females need to use vision only for a brief period of their lives to control flight, to find mating and nesting sites, and to avoid obstacles and predators. Males are also winged and fly out of the nest for mating but in contrast to alate females who merely fly to congregate at a mating site, males actively visually track flying females and competitors and engage in aerial pursuits. In summary, each caste in an ant society performs a unique set of visual tasks and we would expect their visual systems to reflect this.

The visual system of an ant is comprised of a pair of compound eyes and a set of simple eyes called ocelli. Although the compound eyes of worker ants are well studied in some species^1^,^2^,^3^,^4^,^5^ there is surprisingly little known about the visual system of winged ants^6^,^7^. The compound eyes of both pedestrian and flying forms of ants are of an apposition type^7^, each eye is made of several ommatidia, with each ommatidium having its own lens, crystalline cone and photoreceptors. Screening pigments ensheath each ommatidium to ensure light is not shared between neighbouring ommatidia. The pedestrian workers of several ant species rely on the pattern of polarized skylight to obtain compass information^8^. To analyse changes in the polarization pattern ants possess specialized photoreceptors in the dorsal rim area (DRA) of the compound eye. These photoreceptors have orthogonally organised microvilli that enable these ommatidia to encode polarization information^8^,^9^,^10^,^11^,^12^,^13^,^14^. In addition to the compound eye, several species of ants have one to three simple eyes called ocelli on the dorsal surface of the head. Ocelli are extensively referred to in the taxonomic literature, but very little is known about their structure and function and they are thought to be vestigial^15^. In worker ants, ocelli are either absent (most ants) or present in certain castes (e.g., *Gesomyrmex*). When present, there are three ocelli placed in an equilateral triangular formation (e.g., *Cataglyphis, Harpegnathos, Melophorus, Myrmecia*) or in some cases, only the median or the lateral ocelli are present (e.g., *Polyrhachis ypsilon, Polyrhachis bihamata*)^8^. In almost all known ant species, flying males and flying females have three large ocelli^6^,^7^. Behavioural evidence in pedestrian worker ants indicates that ocelli are sensitive to changes in the pattern of polarized skylight^16^ or at least to some celestial cues^17^; furthermore, physiological evidence supports this^18^. Interestingly, recent anatomical evidence in diurnal and nocturnal bees suggests that within each ocellus only a subset of photoreceptors may be sensitive to polarized light^19^. In addition, ocelli could also be involved in sensing terrestrial compass cues, fixating on edges^20^ and detecting the horizon^21^,^22^.

Ants provide us with an opportunity to identify how visual sensory structures have evolved to suit different modes of locomotion and tasks within the same species^23^. There is surprisingly little known about the compound eyes and ocelli of winged ants and of any potential specializations that relate specifically to flight. Here, we study the visual system of the Australian ant, *Camponotus consobrinus* (Erichson), and report on the structure and variation of the compound eyes and the ocelli in the different castes (Fig. 1).

## Results

### Activity schedule and flight time

In the summer months (December-March), foraging activity of workers typically starts 150 minutes before sunset, with peak outbound activity occurring around sunset time (Fig. 2). Most foragers leave the nest before the end of evening astronomical twilight and forage on native Australian trees throughout the night^24^. Majority of the foragers return home during the morning astronomical twilight, with no activity occurring 180 minutes after sunrise. We monitored the time at which alate females and males fly out of the nest over an 8-year period across 12 nests in Canberra that resulted in 62 observation days. Nuptial flights typically occurred between December and March, a day before a rainfall event. Alates departed from the nest in a narrow one-hour period, from 45 minutes before sunset to about 15 minutes after sunset (Fig. 2).

### Variation in head and eye size

**Figure 1:**
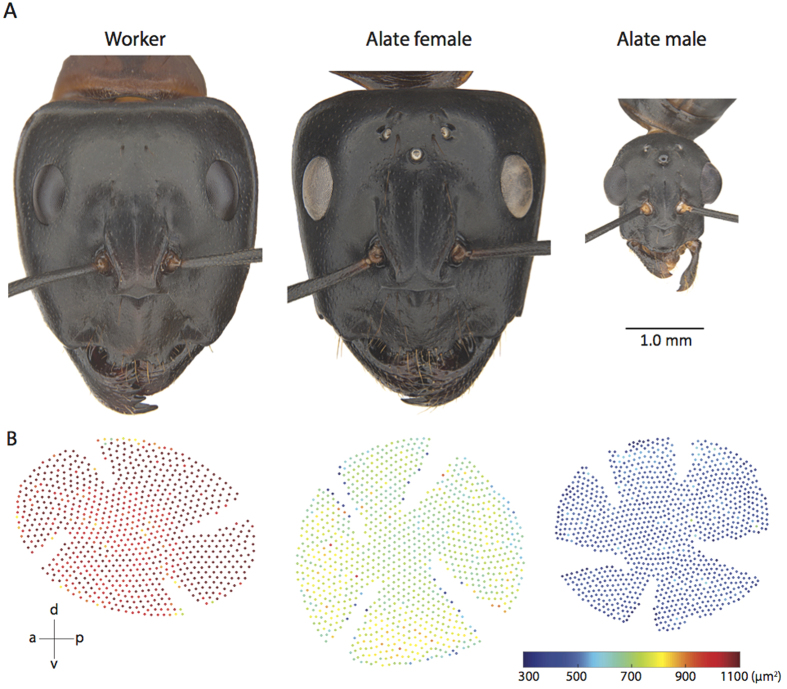
Worker, alate female and male ants of *Camponotus consobrinus*. (**A**) Dorsal micrographs of the head and (**B**) eye maps illustrating facet size and facet distribution.

Males are the smallest of the three castes (head width (HW) = 1.25 ± 0.01 mm, mean ± s.e., n = 6), followed by the workers (3.13 ± 0.001 mm, n = 6) and the alate females (3.25 ± 0.01 mm, n = 6) (p ≪ 0.001, F = 10964, DF = 2,15, ANOVA) (Fig. 1A). Eye length measured along the anterior-posterior axis varied between the three castes with males having the smallest eye size (0.55 ± 0.009 mm), followed by the workers (0.76 ± 0.008 mm) and the alate females (0.80 ± 0.001 mm, n = 6) (p ≪ 0.001, F = 305.3, DF = 2,15, ANOVA, Fig. 3A). Eye length measured along the dorso-ventral axis varied between the three castes, with males having the smallest eye size (0.54 ± 0.002 mm), followed by the workers (0.55 ± 0.007 mm) and the alate female (0.61 ± 0.006 mm, n = 6) (p ≪ 0.001, F = 35.99, DF = 2,15, ANOVA) (Fig. 3A).

### Properties of the compound eye

Facet numbers differ significantly between the three castes with workers having the least number of facets (798.7 ± 18.53; mean ± s.e., n = 6), followed by the alate females (1079.0 ± 26.05, n = 6) and the males (1162.0 ± 13.95, n = 6) (p ≪ 0.001, F = 89.18, DF = 2,15, ANOVA) (Fig. 3B). Lens diameters varied between the three castes, with males having the smallest lens diameter (25.5 ± 0.2 μm, range: 20–29 μm), followed by workers (34.3 ± 0.3 μm, range: 29–41 μm) and the alate females (34.6 ± 0.3 μm, range: 28–40 μm) (p ≪ 0.001, F = 245.5, DF = 2,205, ANOVA) (Figs 1B and 3C). Flying males have the smallest interommatidial angle (4.2°), followed by the flying females (4.4°) and pedestrian workers (5.8°).

The dorsal rim area (DRA) has specialized rectangular or dumbbell-shaped rhabdoms in all three castes. The long axis of these rhabdoms, when viewed in cross-section, are organised in a distinct fan shaped formation (Fig. 4A–C). These rhabdoms are made up of microvilli oriented exclusively in two orthogonal directions compared to the unspecialized rhabdoms that have microvilli oriented in many different directions (Fig. 4A′). The number of specialized DRA photoreceptors varies between castes with the flying males having 90 specialized receptors (Fig. 4C), followed by the alate females that have 86 specialized receptors (Fig. 4B) and the workers with 62 specialized receptors (Fig. 4A). The size of these specialized rhabdoms in cross-section (measured along the long axis) varies between castes with males having the narrowest rhabdoms (2.6 ± 0.05 μm, n = 5 individuals), the alate females being intermediate (4.2 ± 0.05 μm, n = 5 individuals) and the workers having the largest rhabdoms (8.6 ± 0.1 μm, n = 5 individuals) (p ≪ 0.001, F = 1417, DF = 2,55, ANOVA) (Figs 3D and 4). The unspecialized rhabdoms are circular in shape and we measured the diameters of distal rhabdoms in the medio-frontal region of the eye. Here rhabdom diameter varies between castes with males again having the most narrow rhabdoms (1.9 ± 0.03 μm), followed by the alate females (2.8 ± 0.04 μm) and the workers (7.8 ± 0.1 μm) (p ≪ 0.001, F = 1791, DF = 2,44, ANOVA) (Figs 3D and 4). Longitudinal sections of the rhabdom show that the length of the rhabdom in the medio-frontal area of the eye was on average 145.0 μm in workers, 152.2 μm in alate females and 160.8 μm in males (n = 2, for each caste). The focal length in the medio-frontal region of the eye varies from 68.0 μm in workers, 59.6 μm in alate females and 64.5 μm in males. This resulted in a 26-fold difference in optical sensitivity between workers and males (S = 2.7883 μm^2^sr in workers, 0.4838 μm^2^sr in alate females and 0.1068 μm^2^sr in males).

### Properties of the ocelli

Ocelli in *C. consobrinus* are present only in the flying forms, the alate females and males (Figs 1A and 5). Both these castes have three ocelli that are arranged in an equilateral triangle formation on the dorsal surface of the head: a forward facing median ocellus and two ocelli on either side of the midline of the head (Fig. 5). The ant ocellus is comprised of a number of structures starting externally with a thick, convex, corneal lens that has a smooth surface. The ocellar lens is similar in shape in both sexes, but the alate females have a larger ocellar lens compared to the males (alate female: 146.5 ± 0.5 μm, n = 5; males: 101.4 ± 0.7 μm, n = 5, Fig. 5). Proximal to the lens is a vitreous body, followed by a single layer of corneageneous cells, and then the retina (Fig. 5B,C). From longitudinal sections it is clear that the ocellar retina is bipartite with a dorsal retina, which appears to face the horizon and contains retinula cells with long rhabdoms (alate female: 18.6 ± 1.8 μm; males: 22.9 ± 5.2 μm) and a ventral retina, which appears to face the sky and contains retinula cells with shorter rhabdoms (alate female: 9.7 ± 0.3 μm; males: 6.8 ± 2.3 μm). Though alate females of *C. consobrinus* have a large ocellar lens (Fig. 5A) they have only half the number of retinula cells compared to the males (alate female: 242.3 ± 11.2, n = 5 individuals; males: 442.3 ± 28.3, n = 5 individuals, Fig. 6). A cross-section through the ocelli shows that each rhabdom is made up of two retinula cells with microvilli contributing in opposite directions (Fig. 6B,B′). In distal cross-sections of the ocellar retina, the absolute orientation of the rhabdoms varies between the alates, with a majority of the rhabdoms oriented between −18° to + 162° in alate females and −108° to + 126° in alate males (Fig. 7A; 0° = horizontal). Polarization sensitivity of individual receptor cells depends on the straightness of rhabdom in cross-sections^19^. We hence measured rhabdom straightness following the method described by Ribi *et al.*^19^. For this, we divided each rhabdom into four equal segments and calculated the difference between segment orientations and from this the mean orientation of each rhabdom (see Methods for more details). We found that in both sexes, rhabdoms in cross-sections are predominantly straight. In cross-sections, both these crepuscular flying forms of ants had surprisingly narrow rhabdoms (Fig. 7C; alate female: 3.70 ± 0.2 μm; alate male: 3.72 ± 0.2 μm) compared to diurnal honeybees (8.7 ± 2.4 μm) and nocturnal sweat bees (14.3 ± 3.5 μm)^19^.

## Discussion

We have shown here that different castes within a single species have evolved unique visual adaptations, with distinct trade-offs between optical sensitivity, polarization sensitivity and spatial resolution. These adaptations in each caste are finely tuned to suit their specific modes of locomotion, their specific tasks and for the specific light environments they occupy.

**Figure 2:**
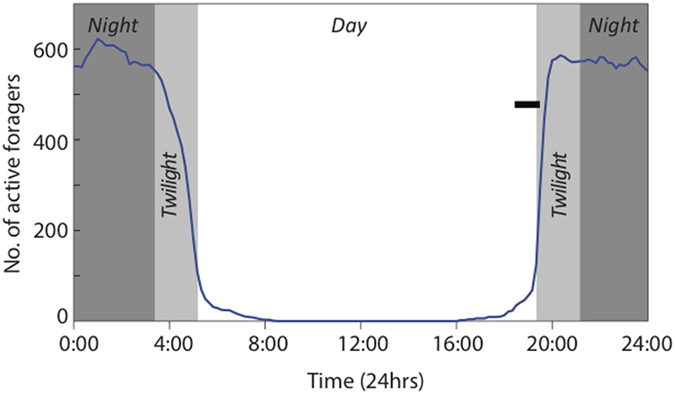
Activity schedule of workers and alates of *Camponotus consobrinus*. Activity pattern of workers on a single summer day is shown in a continuous line. The time at which winged males and females departed from their nest was recorded at 12 nests in Canberra between 2008–2015 with a total of 62 observations. The nest departure time is illustrated by a black horizontal bar.

**Figure 3:**
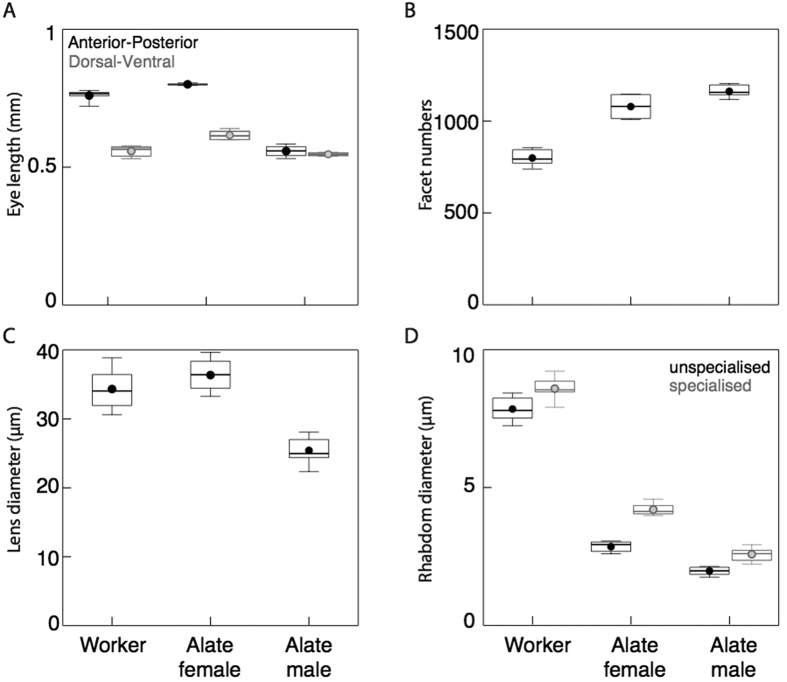
Properties of the compound eye of workers, alate females and males of *Camponotus consobrinus* ants. Box plots show variation in (**A**) eye length, (**B**) facet numbers, (**C**) lens diameter and (**D**) rhabdom diameter of workers, alate females and alate males. Each box plot shows the 75^th^ percentile (top line), 25^th^ percentile (bottom line), median (thick line in the middle), mean (filled circle). Whiskers show the 10^th^ and 90^th^ percentile of the data.

**Figure 4:**
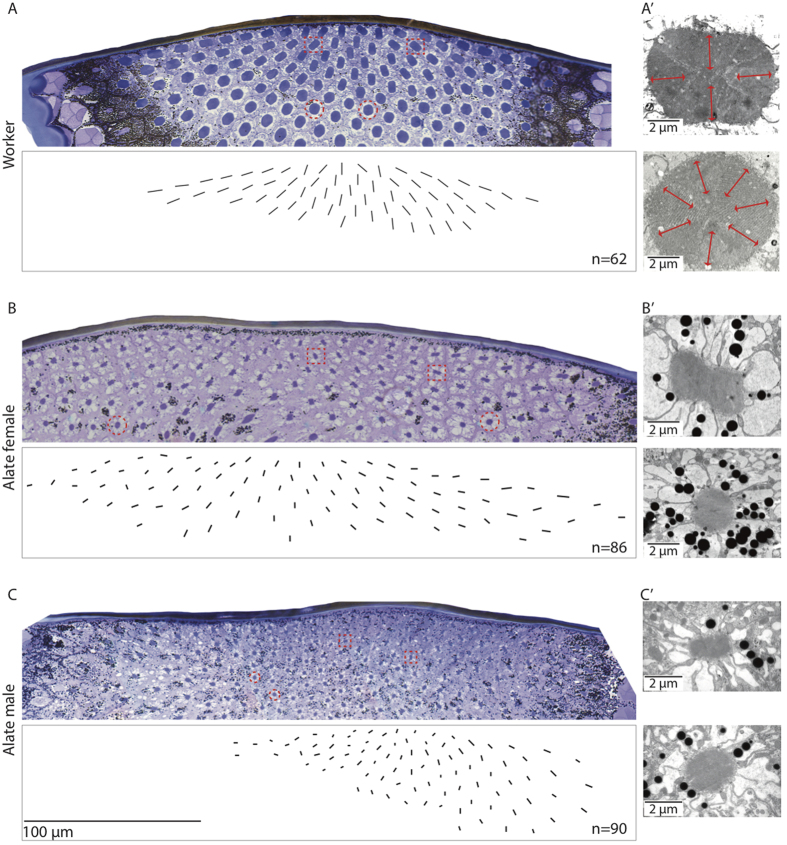
Dorsal rim area showing specialized and unspecialized rhabdoms of worker, alate female and male ants of *Camponotus consobrinus*. Toluidine blue stained light microscopy image (top) and map of the long axis orientations of specialized rhabdoms (bottom) for (**A**) worker, (**B**) alate female and (**C**) alate male are shown. Specialized rectangular rhabdoms (red square) and unspecialized circular rhabdoms (red circle) in light microscopy micrographs are indicated. Transmission electron micrographs of the cross-sectional aspect of the specialized rhabdoms (top) and unspecialized rhabdoms (bottom) for (**A′**) workers, (**B′**) alate females and (**C′**) alate males are shown. Microvilli orientation of a specialized and an unspecialized rhabdom is shown in red lines in (**A′**).

Among the three castes, flying male ants tend to have more and smaller ommatidia and narrow rhabdoms (Fig. 3). Male ants sacrifice optical sensitivity for higher sampling resolution, a trade off that seems to be consistent with the role males play during courtship and their activity time^7^. Males in *C. consobrinus*, in relatively bright light close to the sunset time, actively pursue females and compete with other males for access to queens. To do this they presumably visually track the dark-bodied, flying females against the bright backdrop of the sky, a task that is most demanding on spatial resolution. The size of the lens and rhabdom nicely correlate with activity time (Fig. 2), with lenses being largest and rhabdoms being widest among workers who are active over a larger range of light intensities including much darker conditions than those encountered during twilight. Workers thus increase their optical sensitivity, a 26-fold increase compared to flying males, but have a poorer sampling resolution compared to the males. Similar increase in the optical sensitivity has been documented in nocturnal ants^3^, bees^25^ and wasps^26^. Such trade-offs between optical sensitivity, polarization sensitivity and spatial resolution are also seen in the polarization sensitive dorsal rim area of the eye. In the DRA, the alate males have 90 specialized ommatidia compared to 62 in the pedestrian workers (Fig. 4, see also^27^). But the rhabdom, housed within each ommatidium, is 4 times wider in workers than in the males (Fig. 3D). How this affects an animal’s ability to sense compass information is unclear at this stage: are males, with a greater number of polarization sensitive receptors, or workers, with larger polarization sensitive receptors, more competent in sensing polarization information? Polarization sensitivity increases the contrast between the terrestrial panorama and the sky^8^,^28^. If the observed increase in the number of polarization-sensitive photoreceptors in alates results in an increase in polarization sensitivity, this can potentially contribute additional visual information for controlling the roll, pitch, and yaw movements in flight.

It is only in certain ant species, that pedestrian workers have ocelli. When ocelli are present in worker ants, nocturnal species tend to have larger ocelli compared to their diurnal relatives^6^,^7^, indicating that ocelli may in fact have a visual function and be involved in light capturing. Similarly, ocelli in night-flying male ants (median ocelli diameter: *Atta colombica* = 0.31 mm; *Myrmecia nigriceps* = 0.39 mm) are substantially larger than in their diurnal relatives (median ocelli diameter: *Atta laevigata* = 0.18 mm; *Myrmecia croslandi* = 0.18 mm) again suggesting that ocelli are involved in the visual control of behaviour^6^,^7^. In both bees and wasps it is well known that the night-active animals have larger ocelli than their day-active relatives^29^. In *C. consobrinus*, only flying males and flying females have ocelli, which to the best of our knowledge is the case for all species of *Camponotus* ants. Although the absolute size of the ocellar lens is smaller in males, they are relatively large compared to the alate females (Fig. 1A).

**Figure 5:**
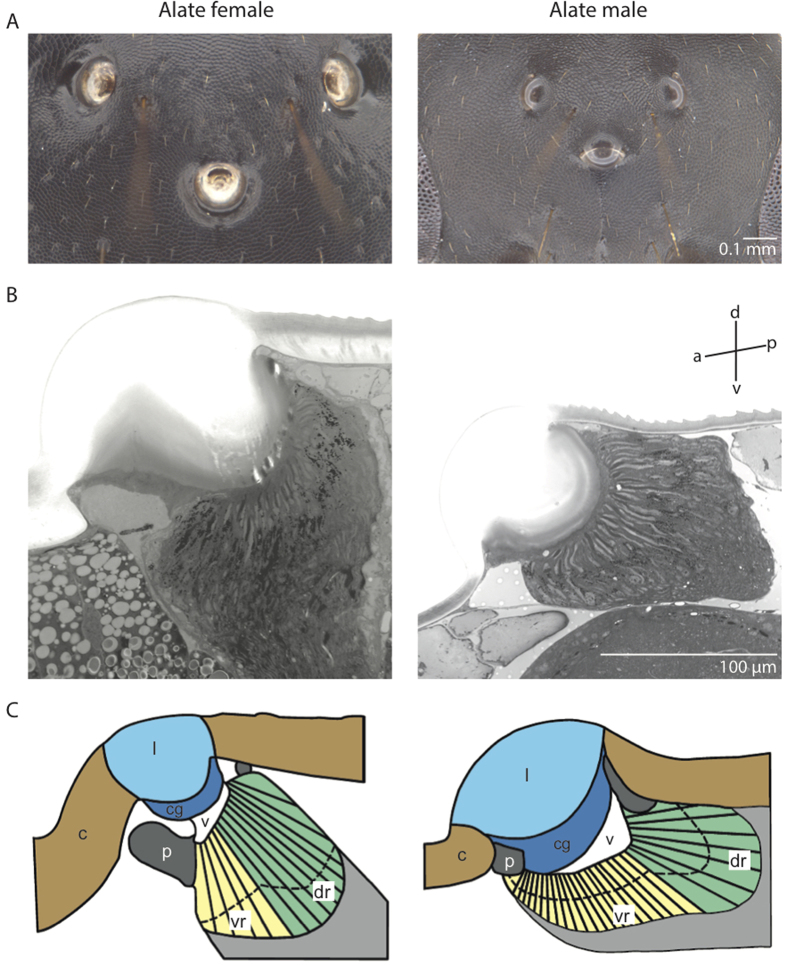
Ocelli of the alate female and male ants of *Camponotus consobrinus*. (**A**) Dorsal micrographs of ocelli, (**B**) longitudinal section of the median ocellus, (**C**) schematic drawing of the longitudinal section of the median ocellus detailing the different optical components: c: cuticle; l: lens; cg: corneagenous layer; v: vitreous body; p: pupil; vr: ventral retina looking at the sky; dr: dorsal retina looking at the horizon. Orientation of the sections and illustrations is shown: a-anterior, p-posterior, d-dorsal and v-ventral.

**Figure 6:**
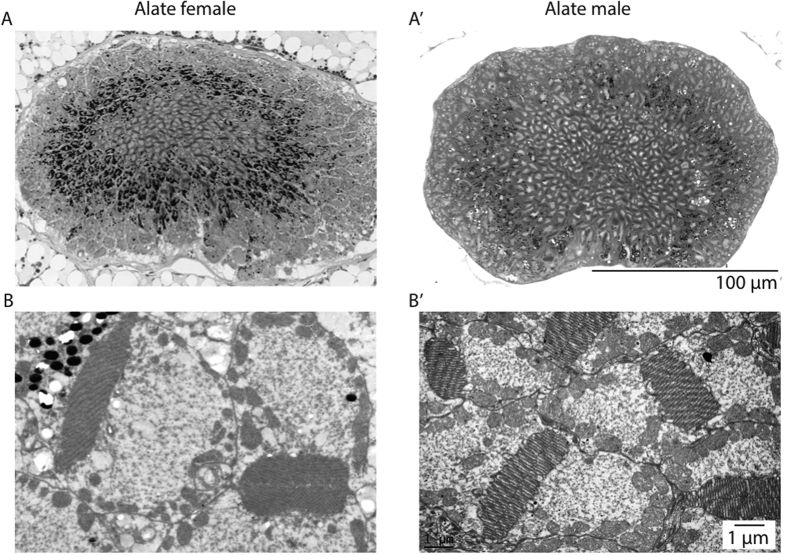
Cross-sections of the median ocellus of the alate female and male ants of *Camponotus consobrinus*. (**A**,**A′**) Light micrographs of cross sections of the median ocellus, (**B**, **B′**) transmission electron micrographs of the cross section of ocellar rhabdoms.

**Figure 7:**
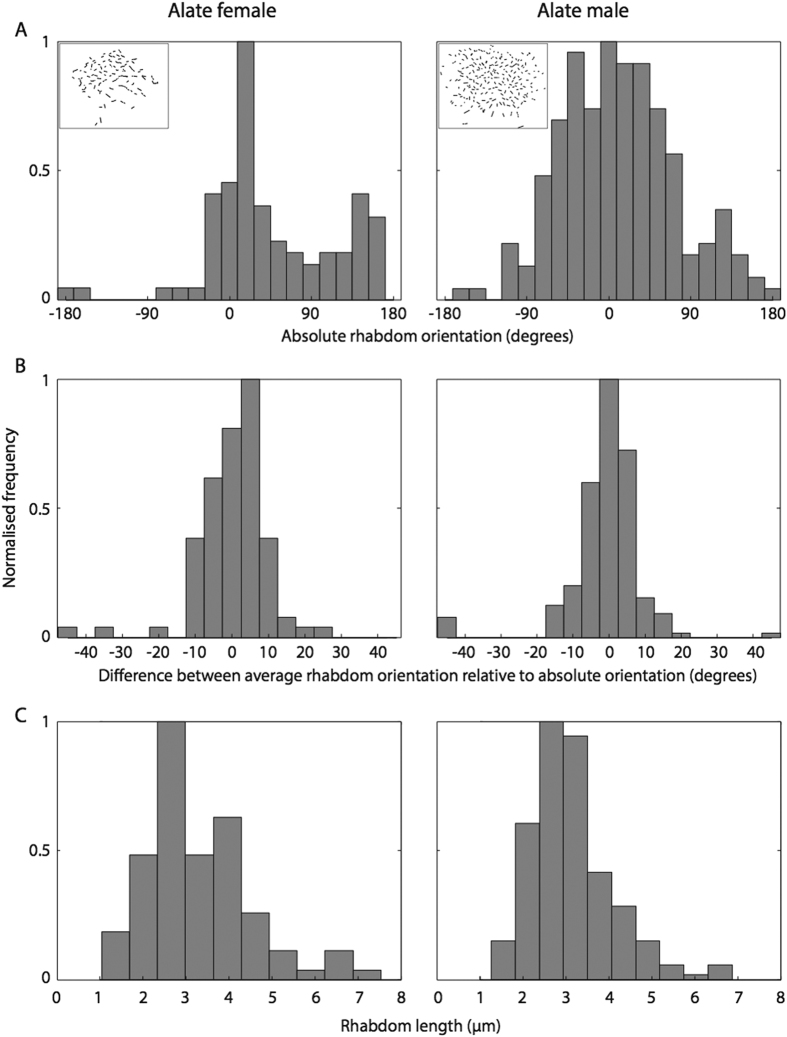
Analysis of ocellar rhabdoms in the median ocellus of alate female and male ants of *Camponotus consobrinus*. (**A**) Histogram of the distribution of absolute rhabdom orientation. Inset: map of rhabdom orientation. (**B**) Histogram of the distribution of the straightness of rhabdoms in cross-sections. Straightness is determined as the difference between the average orientation of all segments (see Methods) relative to absolute rhabdom orientation, with 0° indicating least deviation from the straight line. (**C**) Histogram of the distribution of the length of the rhabdom in cross-sections.

The outer surface of the ocellar lens, in both flying males and females of *C. consobrinus,* is smooth, similar to that of the dragonflies^22^ (Fig. 5B). In contrast, ocellar lenses are bipartite in the worker ant, *Pheidologeton diversus*^15^ and in the honeybee, where one-third of the lens is directed dorsally and two-thirds of the lens is directed frontally (in the median ocellus) or ventrally and laterally (in the lateral ocelli)^19^. Ocelli in male ants tend to be closer to each other compared to the situation in flying females (Fig. 1A). This may be simply because male ants have smaller heads. It is crucial to identify in future studies whether the three ocelli in the alates allows for an overlap of the dorsal and the frontal ocellar visual fields, and how this compares with workers when they have ocelli. While in several insects ocellar lenses under-focus the light onto the retina, recent evidence in dragonflies^22^, in wasps^29^ and in honeybees^19^ suggests that the ocellar retina may be able to resolve images.

The ocellar retina in both flying males and flying females in *C. consobrinus* are bipartite (Fig. 5B,C). The dorsal retina appears to be directed towards the horizon, an adaptation for attitude control during flight. The ventral retina appears to be directed towards the sky, perhaps an adaptation to receive skylight, as has been suggested in the honeybees^19^. This opens up the possibility that the pattern of polarised skylight may be captured by the ventral retina. Such a bipartite retina is well developed in other exclusively flying hymenopterans^8^,^19^. Currently, we know very little about the function and structure of the ocelli in ants. There is some evidence in the diurnal desert ant, *Cataglyphis bicolor*, that ocelli are involved in detecting changes in the pattern of polarized skylight^16^,^18^, but there have been no descriptions of the ocellar anatomy or underlying neural circuitry. In the workers of the nocturnal bull ant, *Myrmecia pyriformis*, the ocellar retina is well developed but rhabdom orientation has a very large distribution (−145° to +60°)^8^, and very few rhabdoms are straight^8^, suggesting that only few receptor cells are polarization sensitive. Ocelli are largest and most prominent in the flying ants^6^,^7^, their rhabdoms are straight and are comparable to other flying hymenopterans such as *Amegilla holmessi*, *Apis mellifera*, *Sphex cognatus*^8^. Flying ants also seem to have several ocellar retinula cells that are sensitive to polarized light and hence, there is a need to quantify polarization sensitivity of the ocelli in alate ants and identify how this assists in their behavioural tasks.

## Methods

### Study species

We studied the structure of the compound eye and ocelli of the Australian Banded Sugar ant, *Camponotus consobrinus*. On a cloud-free summer day in Canberra, we monitored the outbound and inbound activity of ants at one nest for a 24-hr period. A more detailed activity schedule of these ants is presented elsewhere^24^,^27^. Between 2008–2015 we monitored 12 nests and recorded the time at which winged forms left the nest. To investigate the visual system, we collected workers from multiple nests found on The Australian National University campus, Canberra. Workers of *C. consobrinus* are polymorphic and the relation between the number of ommatidia and worker size has been recently described^27^. We focused our investigation on workers of the largest size (head width (HW) = 3.13 ± 0.01 mm). Alate females (HW = 3.23 ± 0.03 mm) and males (HW = 1.25 ± 0.03 mm) are monomorphic (Fig. 1A). For overview images, specimens were mounted and imaged in a Leica M205C stereomicroscope with a DFC 500 colour camera. Z-stacks were merged using either Leica LAS software or Zerene Stacker (v1.04, copyright 2009–2014 Zerene Systems, LLC).

### Facet numbers, size and distribution

We covered the compound eyes with a thin layer of colourless nail polish to produce cornea replicas^30^,^31^. Once dry, the replicas were carefully removed and flattened on a microscope slide by making incisions with a micro-scalpel. The replicas were photographed in a Zeiss light microscope. We determined facet numbers and lens diameters of six individuals for each caste. We used one replica for each caste to map the facet area and distribution using a custom-written program in Matlab (© Richard Peters, La Trobe University). The ocellar lens is highly convex and hence is therefore difficult to reliably measure lens diameters directly from either specimens or from SEM images. We hence measured the ocellar lens dimensions from longitudinal sections.

### Histology

Ants were sedated by cooling on ice, their mandibles were removed and head capsules were opened. For processing the eyes, optimal fixation was achieved by removing the most ventral rim of the eye. For processing the ocelli, optimal fixation was achieved by making incisions in the anterior and posterior region of the head closest to the ocelli. Specimens were fixed for four hours in a volume of 2.5% glutaraldehyde and 4% paraformaldehyde in phosphate buffer (pH 7.2–7.5). This was followed by a series of buffer washes and post-fixation in 2% OsO_4_ solution in distilled water for one hour. Samples were then dehydrated in an ethanol series, transferred to propylene oxide and embedded in Epoxy resin (FLUKA). We made semi-thin (1 μm) and ultra-thin (40–60 nm) sections of both the ommatidia and ocelli on a UC7 EM Leica Ultra microtome using a diamond knife (DIATOME). The semi-thin sections for light microscopy were stained with toluidine blue and digitally photographed in a Zeiss Axioskop. The ultra-thin sections for transmission electron microscopy were stained with 6% saturated uranyl acetate (25 min) and lead citrate (5 min) before viewing with a transmission electron microscope (Hitachi HA7100 TEM or Philips FEI CM300 TEM).

### Analysis

High magnification images of the dorsal rim area cross-sections were manually aligned and stitched. Images of cross-sections (both DRA and ocelli) were imported and specific positions on the rhabdom were digitized using a custom-written software Digilite (© Jan Hemmi & Robert Parker) in Matlab (Mathworks, Nattick, USA) to extract x/y coordinates. The two furthest positions of each rhabdom were digitized and the orientation of these two coordinates provided a rhabdom’s absolute orientation. For ocellar rhabdoms, we digitised five equidistant points along the long axis of each rhabdom. We then determined the ocellar rhabdom straightness in the direction of the long axis. For this, we measured the orientation of the four segments (point 1–2, 2–3, 3–4, 4–5) and calculated the difference between the segment orientation and the absolute orientation (i.e., point 1–5), a method used to study honeybee ocellar rhabdom^19^. We determined the rhabdom length, by summing the length of all four segments for each rhabdom.

We calculated the interommatidial angle, ∆ø, by assuming each eye has a hemispherical visual field^11^,


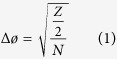


where, Z = Sphere = 41252.96 square degrees; N = facet number.

This method is an approximation and an accurate measure of interommatidal angle for the entire eye can be obtained by mapping the pseudopupil of the eye^23^.

We determined the optical sensitivity, S, which is the sensitivity of an eye to an extended scene of broad spectral content as follows^32^:


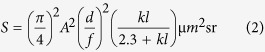


where, A = largest lens diameter (μm); d = diameter of the rhabdom (μm); f = focal length, determined by the distance from the centre of curvature of the inner corneal lens surface (as an estimate for the position of the nodal point) to the tip of the rhabdom; l = the rhabdom length; k = absorption coefficient assumed to be 0.0067μm^−1 ^ ref. 33.

## Additional Information

**How to cite this article**: Narendra, A. *et al.* Compound eye and ocellar structure for walking and flying modes of locomotion in the Australian ant, *Camponotus consobrinus. Sci. Rep.* **6**, 22331; doi: 10.1038/srep22331 (2016).
